# Synthesis and structural characterization of the dichloride complex formed by carb­oxy-functionalized Cu(di­aza­cyclam)^2+^ cation and its heterometallic coordination polymer with CdCl_2_

**DOI:** 10.1107/S2056989025003792

**Published:** 2025-05-02

**Authors:** Liudmyla V. Tsymbal, Irina L. Andriichuk, Alexandru-Constantin Stoica, Yaroslaw D. Lampeka

**Affiliations:** a L. V. Pisarzhevskii Institute of Physical Chemistry of the National Academy of Sciences of Ukraine, Prospekt Nauki 31, 03028, Kyiv, Ukraine; b "Petru Poni" Institute of Macromolecular Chemistry, Aleea Gr. Ghica Voda 41A, RO 700487, Iasi, Romania; University of Aberdeen, United Kingdom

**Keywords:** crystal structure, di­aza­cyclam, 3-carb­oxy­propyl substituent, copper, cadmium, coordination polymer, hydrogen bonds.

## Abstract

The asymmetric unit of the complex [3,10-bis­(3-carb­oxy­prop­yl)-1,3,5,8,10,12-hexa­aza­cyclo­tetra­decane-κ^4^*N*^1^,*N*^5^,*N*^8^,*N*^12^]di­chlorido­copper(II), [CuCl_2_(C_16_H_34_N_6_O_4_)] or [Cu(H_2_*L*)Cl_2_] (**I**), consists of a centrosymmetric macrocyclic Cu^II^ dication and a chloride anion. The components of the heterometallic compound poly[[aqua­[μ_3_-3,10-bis­(3-carb­oxy­prop­yl)-1,3,5,8,10,12-hexa­aza­cyclo­tetra­dec­ane-κ^4^*N*^1^,*N*^5^,*N*^8^,*N*^12^:κ^2^*O*,*O*′:κ^2^*O*′′,*O*′′′]-μ-chlorido-­copper(II)cadmium(II] 1.25-hydrate], {[CuCd(C_16_H_32_N_6_O_4_)Cl_2_(H_2_O)]·1.25H_2_O}_*n*_ or {[CuCd(*L*)(H_2_O)Cl_2_]·1.25H_2_O}_*n*_ (**II**) are [Cu(*L*)(H_2_O)] moieties coordinated to CdCl_2_ units *via* the deprotonated carb­oxy­lic groups of the macrocycle, and four water mol­ecules of crystallization with partial occupancies. In each compound, the Cu^II^ ion coord­inates in the equatorial plane by the four secondary N atoms of the macrocyclic ligand, which adopts the most energetically stable *trans*-III conformation, and two mutually *trans* axial ligands in tetra­gonally elongated *trans*-CuN_4_Cl_2_ and *trans*-CuN_4_(H_2_O)Cl octa­hedral geometries in **I** and **II**, respectively. The coordination environment of the Cd^II^ ion in **II** is a CdO_4_Cl_2_ distorted octa­hedron formed by two bidentately coordinated deprotonated carb­oxy­lic groups of different macrocycles and two chloride anions, one of which displays a μ_2_-bridging function between the Cu^II^ and Cd^II^ ions. The extended structures of both complexes are distinctly lamellar. In particular, due to hydrogen–bonding inter­actions with participation of carb­oxy­lic groups, chloride atoms and secondary amino groups of the macrocycle, the electro-neutral mol­ecules in crystal of **I** are arranged in chains running in the [101] direction; hydrogen bonding between the chains leads to layers parallel to the (101) plane. In crystal of **II**, polymeric chains running along the [101] direction are joined into layers parallel to the (101) plane *via* formation of Cu—Cl bonds and inter­chain hydrogen bonds. There are no hydrogen-bonding inter­actions between the layers and the three-dimensional structure of **II** is based on the weak C—H⋯O and C—H⋯Cl contacts.

## Abstract 2

The coordination polyhedra of the Cu^II^ ions in mol­ecular complex **I** and coordination polymer **II** represent tetra­gonally elongated *trans*-CuN_4_Cl_2_ and *trans*-CuN_4_(H_2_O)Cl octa­hedra, respectively, with the four N atoms of the macrocyclic ligand forming the equatorial plane and monodentate ligands occupying the axial positions. The coordination polyhedron of the Cd^II^ ion in **II** is a CdO_4_Cl_2_ distorted octa­hedron formed by two bidentately coordinated deprotonated carb­oxy­lic groups of different Cu^II^ macrocyclic cations and two chloride anions, one of which displays a bridging function.

## 1. Chemical context

Owing to exceptionally high thermodynamic stability and kinetic inertness (Yatsimirskii & Lampeka, 1985 ▸), first row transition-metal complexes of the tetra­dentate 14-membered aza­macrocyclic ligand 1,4,8,11-tetra­aza­cyclo­tetra­decane (cyclam) and its N^3^,N^10^-disubstituted structural analogue 1,3,5,8,10,12-hexa­aza­cyclo­tetra­decane (di­aza­cyclam) are common metal-containing nodes for the design of metal–organic frameworks (MOFs), demonstrating many promising applications (Lampeka & Tsymbal, 2004 ▸; Suh & Moon, 2007 ▸; Stackhouse & Ma, 2018 ▸). The Ni^II^ and Cu^II^ complexes of di­aza­cyclam are readily obtainable *via* template Mannich condensation of bis­(ethyl­enedi­amine) complexes of these cations with formaldehyde and primary amines (Costisor & Linert, 2000 ▸). The use of primary amines bearing an additional coordinating groups as locking fragments in these template reactions allows for the preparation of complexes of functionalized di­aza­cyclams. As a result of the inter­action of the donor group of the substituents in these species with other metal-containing nodes they can form coordination polymers, without using additional bridging ligands. Indeed, several examples of polymeric compounds formed by the Ni^II^ or Cu^II^ complexes of propio­nitrile-substituted di­aza­cyclam have been described (Suh *et al.*, 1994 ▸; Liu *et al.*, 2002 ▸). They are the homometallic products of self-polymerization reactions occurring *via* coordination of the nitrile groups of the substituents of macrocyclic cation in the axial positions of the metal ions of other macrocyclic units. Similar self-polymerization of building blocks is also characteristic of 3-carb­oxy­propyl-substituted di­aza­cyclam (Lu *et al.*, 2005 ▸; Ou *et al.*, 2005 ▸ see *Database survey*). Because carboxyl­ates are known as the most popular bridging units in preparation of MOFs (Rao *et al.*, 2004 ▸; Yoshinari & Konno, 2023 ▸), the complexes of this ligand are of particular inter­est because their reactions with other metal ions can lead to formation of new types of heterometallic coordination polymers.
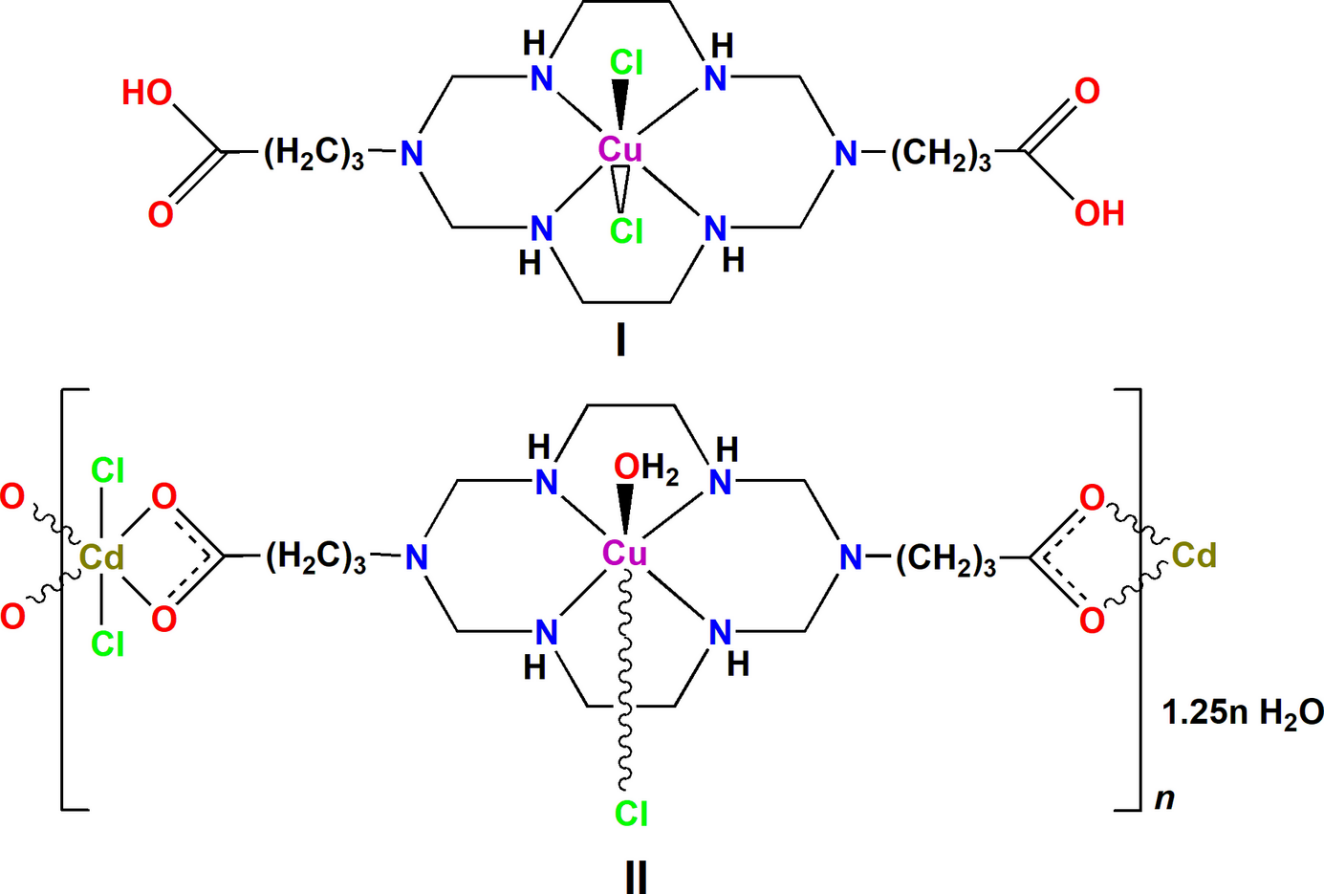


The present work describes the preparation and structural characterization of the mol­ecular Cu^II^ dichloride complex of the aza­cyclam ligand 3,10-bis­(3-carb­oxy­prop­yl)-1,3,5,8,10,12-hexa­aza­cyclo­tetra­decane (H_2_*L*), namely, [3,10-bis­(3-carb­oxy­prop­yl)-1,3,5,8,10,12-hexa­aza­cyclo­tetra­decane-κ^4^*N*^1^,*N*^5^,*N*^8^,*N*^12^]di­chlorido­copper(II), [CuCl_2_(C_16_H_34_N_6_O_4_)] or [Cu(H_2_*L*)Cl_2_] (**I**), and the product of its inter­action with Cd^II^ ion, namely, poly{[aqua­[μ_3_-3,10-bis­(3-carb­oxy­prop­yl)-1,3,5,8,10,12-hexa­aza­cyclo­tetra­dec­ane-κ^4^*N*^1^,*N*^5^,*N*^8^,*N*^12^;κ^2^*O*,*O*′:κ^2^*O*′′,*O*′′′]-μ-chlorido­copper(II)cadmium(II] 1.25-hydrate], {[CuCd(C_16_H_32_N_6_O_4_)Cl_2_(H_2_O)]·1.25H_2_O}_*n*_ or {[CuCd(*L*)(H_2_O)Cl_2_]·1.25H_2_O}_*n*_ (**II**), which is the first representative of heterometallic polymeric complexes with carboxyl-substituted Cu-di­aza­cyclam moiety as bridging ligand.

## 2. Structural commentary

**Figure 1:**
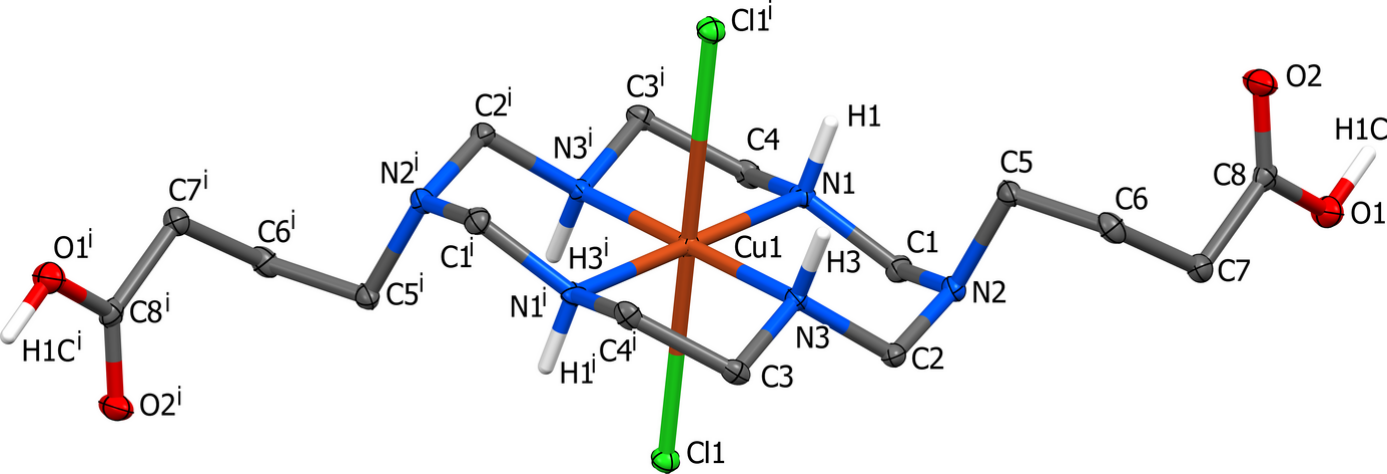
The asymmetric unit in **I** showing displacement ellipsoids drawn at the 30% probability level. C-bound H atoms are omitted for clarity. Symmetry code: (i) −*x* + 2, −*y* + 1, −*z* + 1.

**Figure 2:**
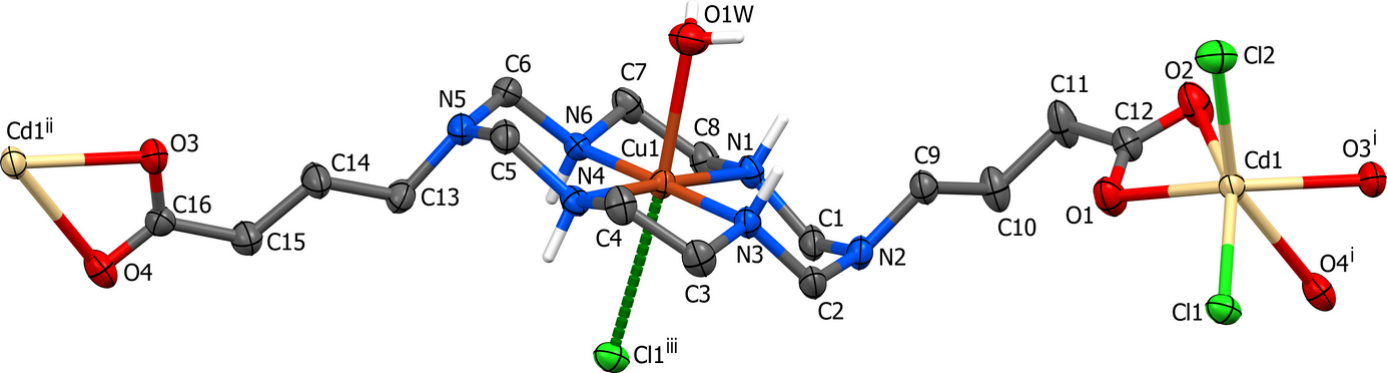
The extended asymmetric unit in **II** with displacement ellipsoids drawn at the 30% probability level. The semicoordinative Cu—Cl1 bond is shown as dark green capped stick line. C-bound H atoms are omitted for clarity. Water mol­ecules of crystallization are not shown. Symmetry codes: (i) *x* + 1, *y*, *z* + 1; (ii) *x -* 1, *y*, *z* − 1; (iii) −*x* + 1, −*y*, −*z* + 1.

The mol­ecular structures of **I** and **II** are shown in Figs. 1 ▸ and 2 ▸, respectively, while selected geometric parameters characterizing the coordination environment of the Cu^II^ and Cd^II^ ions are collected in Tables 1 ▸ and 2 ▸. The asymmetric unit of **I** (Fig. 1 ▸) represents a half of the neutral centrosymmetric [Cu(H_2_*L*)Cl_2_] complex formed by a di­aza­cyclam ligand with protonated carb­oxy­lic groups. The asymmetric unit of **II** contains a [Cu(*L*)(H_2_O)] moiety coordinated to CdCl_2_*via* deprotonated carb­oxy­lic groups of the macrocycle (Fig. 2 ▸). Additionally, it includes four water mol­ecules of crystallization with site occupancies 0.5 (O2*W*) and 0.25 (O3*W*–O5*W*) (total 1.25 water mol­ecules).

In both compounds the Cu^II^ ion coordinates the four secondary N atoms of the macrocycles, which adopt the most energetically stable *trans*–III (*R,R,S,S*) conformation (Barefield *et al.*, 1986 ▸) with the five-membered (N—Ni—N bite angles *ca* 86°) and six-membered (N—Ni—N bite angles *ca* 94°) chelate rings adopting *gauche* and *chair* conformations, respectively (Tables 1 ▸ and 2 ▸). The macrocyclic ligands are in stretched forms with remote carb­oxy­lic groups. At the same time, the noticeable difference in the distances between their C atoms [C8—C8(–*x* + 2, –*y* + 1, –*z* + 1) of 14.241 (6) Å and C12—C16 of 15.29 (1) Å in **I** and **II**, respectively] is caused by different conformations of the tri­methyl­ene fragments of the substituents. The methyl­ene groups bound to the distal nitro­gen atoms in the six-membered chelate rings are axially oriented. Therewith, the sum of the C—N—C angles around these atoms [345.0° for N2 in **I** and 347.8 and 351.2° for N2 and N5 in **II**, respectively] indicates their partial *sp*^2^ character (Tsymbal *et al.*, 2019 ▸). The C—O bond lengths in the protonated carb­oxy­lic group in **I** differs significantly [1.319 (4) and 1.204 (3) Å for the C—OH and C=O bond, respectively], while in **II** they are nearly identical (*ca* 1.24 Å), thus indicating the lack of electron delocalization in the former and its occurrence in the latter case.

The aza­macrocyclic ligands in the complexes under consideration are coordinated to the Cu^II^ ions by four secondary amine N atoms in a planar fashion forming the equatorial planes in the coordination spheres of the metal ions. The axial positions are occupied by the two chloride anions (in **I**) or by the O atom from the water mol­ecule and chloride anion belonging to another mol­ecule (in **II**). Because of a large Jahn–Teller distortion inherent in the 3*d*^9^ electronic configuration of Cu^II^, the equatorial Cu—N bonds are significantly shorter than the axial Cu—Cl and Cu—O ones (Tables 1 ▸ and 2 ▸), therefore the coordination polyhedra can be described as tetra­gonally elongated *trans-*CuN_4_(Cl)_2_ or *trans-*CuN_4_(O)(Cl) octa­hedrons in **I** and **II**, respectively. The length of the Cu—Cl bond in **I** is close to those observed in other Cu^II^–chloride complexes of di­aza­cyclam ligands (see *Database survey*). At the same time, the distance Cu1—Cl1(−*x* + 1, −*y*, −*z* + 1) [3.048 (2) Å] in **II** is significantly longer. Nevertheless, it is shorter than the sum of van der Waals radii of these atoms (3.15 Å) thus allowing to consider this Cu—Cl contact as a week coordinative [or semicoordinative (Valach, 1999 ▸)] bond.

The CuN_4_ moiety in **I** is strictly planar because of the location of the metal ion on crystallographic inversion center, while in **II** the Cu^II^ ion is displaced by 0.03 Å from the nearly planar (deviations of ±0.015 Å) mean plane of the N_4_ donor atoms towards the O1*W* atom of water mol­ecule. The axial Cu—*D* bonds (*D* = donor atom) in both compounds are nearly orthogonal to the CuN_4_ plane with the deviations of the angles N—Cu—*D* from the normal not exceeding 5°.

The coordination polyhedron of the six-coordinated Cd^II^ ion in **II** is formed by the two bidentately coordinated carb­oxy­lic groups and two chloride ions. The metal ion possesses a deformed octa­hedral environment with *cis*-situated chloride anions. The values of chelate bite angles of the four-membered chelate rings are determined by the geometrical parameters of the coordinated carboxyl­ate groups and analogously to other Cd^II^ carboxyl­ate complexes (see, for example, Popovych *et al.*, 2024 ▸*)* are close to 53° (Table 2 ▸). The angle between the mean planes of these chelate rings is 87.8 (2)°. The Cd—Cl bond lengths in **II** are very similar and are longer than the Cd—O bonds which, in turn, are significantly non-equivalent within each carboxyl­ate group (Table 2 ▸).

The macrocyclic di­carboxyl­ate complex anion in **II** displays a bridging function between two Cd^II^ cations. Each metal ion coordinates the carboxyl­ate groups of two different macrocyclic ligands, thus resulting in the formation of a linear (the angle Cd⋯Cd⋯Cd = 180°) coordination polymeric chain, running along the [101] direction, with the shortest intra­chain Cd^II^⋯Cd^II^ (and Cu^II^⋯Cu^II^) distances of 19.2082 (8) Å. The distances between hetero metal ions equal 9.296 (1) and 10.414 (1) Å for Cu1⋯Cd1 and Cu1⋯Cd1(*x* − 1, *y*, *z* − 1), respectively (Fig. 2 ▸).

## 3. Supra­molecular features

**Figure 3:**
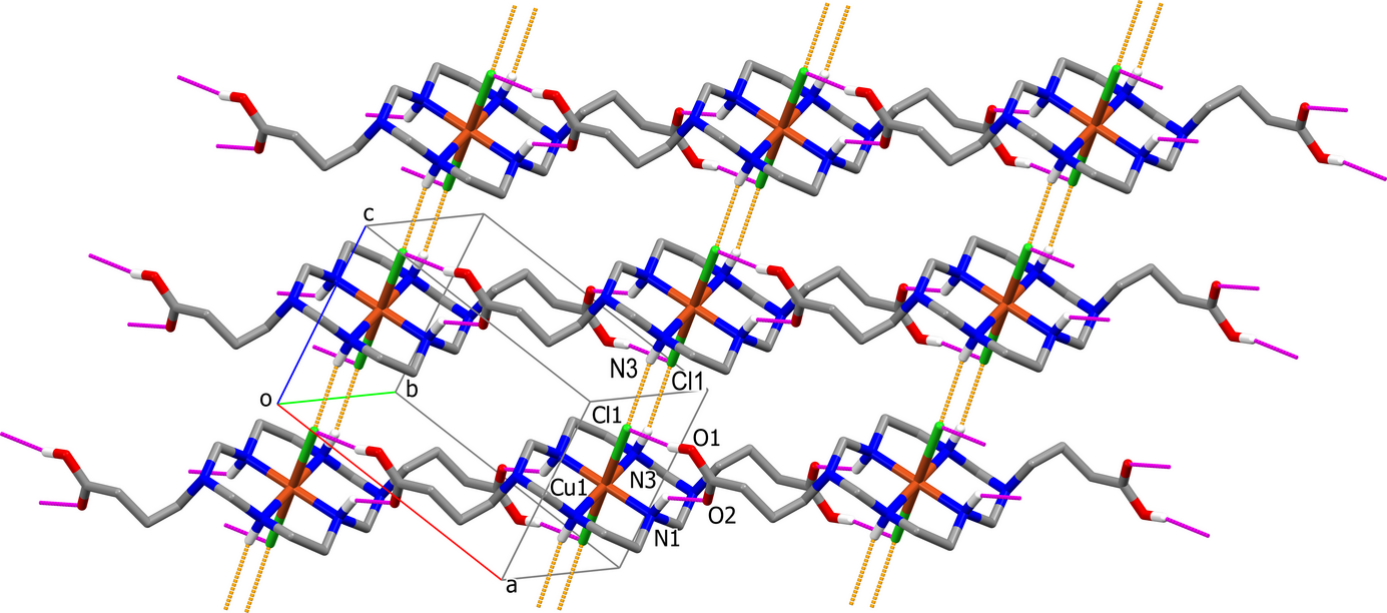
The hydrogen-bonded layer in **I** oriented parallel to the (010) plane. C-bound H atoms have been omitted. The hydrogen bonds resulting in the formation of one-dimensional chains and those joining them into a layer are shown as magenta and orange dashed lines, respectively.

The complex **I** is characterized by a distinct lamellar structure, which is due to hydrogen-bonding inter­actions (Table 3 ▸). In particular, each carboxyl­ate group of the electro-neutral complex mol­ecule forms a pair of hydrogen bonds to a neighboring one acting as the proton donor for the chloride ion [O1C O1—H1*C*⋯Cl1(*x* − 1, *y*, *z* − 1)] and as the proton acceptor for the secondary amino group [N1—H1⋯O2(−*x* + 1, −*y* + 1, −*z*)]. This results in the formation of chains running in the [101] direction, with the distance between the metal atoms in a chain equal to 11.1582 (5) Å. These chains inter­act with each other *via* the formation of a hydrogen bond between another secondary amino group of the macrocycle and a chloride anion [N3—H3⋯Cl1(*x*, *y*, *z* − 1)], thus leading to layers oriented parallel to the (101) plane (Fig. 3 ▸). The shortest inter­chain Cu⋯Cu distance is 6.9325 (3) Å. There are no hydrogen-bonding contacts between the layers and the three-dimensional coherence of the crystal is provided by van der Waals inter­actions.

**Figure 4:**
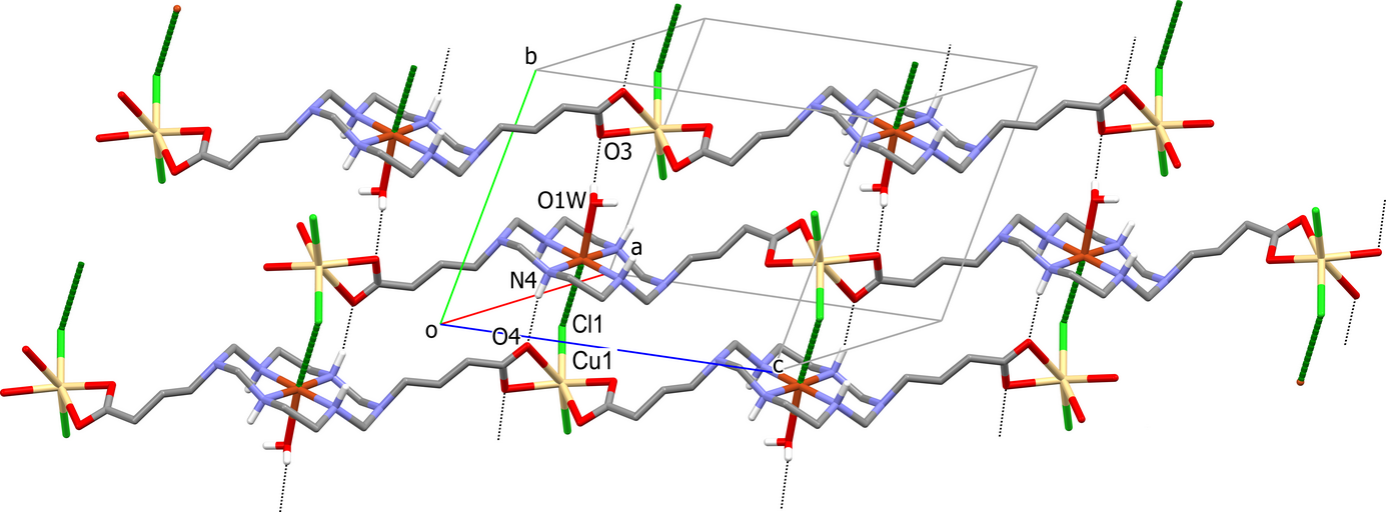
The packing in **II**, showing [101] polymeric chains cross-linked by Cu—Cl semicoordinative bonds (dark-green capped stick lines) to form layers oriented parallel to the (10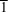) plane. C-bound H atoms are omitted for clarity. Intra­layer hydrogen bonds are shown as dashed lines. Hydrogen bonds with participation of chloride anion are not shown.

The parallel polymeric chains in the crystal of **II** inter­act with each other *via* the formation of semicoordinative Cu—Cl1(−*x* + 1, −*y*, −*z* + 1) bonds between atoms belonging to neighboring chains thus joining them into layers oriented parallel to the (10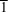) plane (Fig. 4 ▸). Thus, atom Cl1 in this compound displays a μ_2_-bridging function with a Cu⋯Cd distance and Cu—Cl—Cd angle of 4.807 (1) Å and 119.33 (6)°, respectively. The layers are further consolidated by inter­chain hydrogen bonds formed between coordinated water mol­ecule O1*W*—H and secondary amino group N4—H as the proton donors and both O atoms of the C16/O3/O4 carb­oxy­lic group as the proton acceptors (Fig. 4 ▸), as well as by weaker (*D*⋯*A* distances *ca* 3.4 Å) hydrogen bonds with participation of both chloride atoms as proton acceptors (Table 4 ▸). The intra­layer hydrogen-bonding network also includes the water mol­ecule of crystallization O2*W* (because of the lower site occupancy factors of O3*W*–O5*W* mol­ecules their participation in the hydrogen-bonding inter­actions is not considered). There are no significant hydrogen-bonding inter­actions between the layers and the three-dimensional structure of crystal **II** is based on the weak C—H⋯O and C—H⋯Cl contacts.

## 4. Database survey

The Cambridge Structural Database (CSD, version 5.46, September 2024; Groom *et al.*, 2016 ▸) contains four structures formed by di­aza­cyclam ligand *H_2_L* with Ni^II^ [CSD refcodes NARBAK and NARBEO (Lu *et al.*, 2005 ▸)] and Cu^II^ [WAMWEN and WAMWIR (Ou *et al.*, 2005 ▸)] ions. Besides, the structure of the Cu^II^ complex of parent monosubstituted aza­cyclam ligand 3-(3-carb­oxy­prop­yl)-1,3,5,8,12-penta­aza­cyclo­tetra­decane [NUBFOI (Tsymbal *et al.*, 2019 ▸)] has been also described. Among these compounds, NARBAK and WAMWEN represent *trans*-di­aqua mol­ecular complexes with the macrocycle *L^2–^* bearing deprotonated but uncoordinated carb­oxy­lic groups. NUBFOI and WAMWIR are one- and two-dimensional coordination polymers, respectively, which are the result of self-polymerization due to the coordination of the carb­oxy­lic groups to the metal ion of another mol­ecules. Inter­estingly, in both cases the Cu^II^ ion is bound with the carbonyl O atom of a protonated carboxyl­ate group. In NARBEO the ligand *H_2_L* is also present in protonated form and the charge of the cation is compensated by deprotonated but-2-enedioate which acts as bridge between the Ni^II^ centres thus resulting in the formation of a one-dimensional coordination polymer without involving in polymerization the carb­oxy­lic groups of the aza­cyclam ligand. Despite differences in the nature of the metal ions and protonation peculiarities, the macrocycles in all compounds possess very similar stretched *trans*-III (*R,R,S,S*) conformations with the distances between carb­oxy­lic groups varying between 13.6–15.0 Å, so this distance in **II** is the longest among all complexes studied.

There are only two examples in the CSD concerning the structure of *trans*-dichloride complexes of Cu(di­aza­cyclam)^2+^ cations with 2-hydroxyehyl [MANKOB (Lampeka *et al.*, 1998 ▸)] and ethyl [MEDRAP (Jiang *et al.*, 2006 ▸)] substituents at distal nitro­gen atoms of the macrocycle. In both cases the Cu—Cl coordination bond lengths are close to that observed in **I** (*ca* 2.83 Å), regardless of whether the chloride ion demonstrates monodentate (MEDRAP) or bridging (MANKOB) function. Similar distances are also observed in Cu^II^ chloride complexes of cyclam [see, for example, FODWAZ (Samoľová *et al.*, 2019 ▸) and QASKUU (Heinemann *et al.*, 2022 ▸)], though a much shorter Cu—Cl coordination bond was also found [2.446 (4) Å in YEGMEF (Chang *et al.*, 2017 ▸)].

The CSD contains also six hits related to the six-coordinated Cd^II^ complexes containing two bidentately coordinated carboxyl­ate ligands and two chloride anions. Four of them represent mol­ecular compounds formed by substituted propionic [NASWUZ (Li & Mak, 1997 ▸); VOGKUZ (Galkina *et al.*, 2014 ▸); UBILOK (Yang *et al.*, 2021 ▸)] or benzoic [VIQLIR (Deng *et al.*, 2007 ▸)] acids, while two other are one-dimensional coordination polymers based on di­carboxyl­ates [KESGEX (Liu *et al.*, 2017 ▸); KIRFAW (Jin *et al.*, 2023 ▸)]. Regardless of the structure, the geometrical parameters of the coordination polyhedra of the Cd^II^ ion in these compounds are very similar and resemble those observed in **II**.

## 5. Synthesis and crystallization

All commercially available chemicals and solvents were used in this work as purchased without further purification. The complex [Cu(*H_2_L*)]Cl_2_·2H_2_O was synthesized according to a procedure described previously (Ou *et al.*, 2005 ▸).

The complex **[Cu(*****H_2_L*****)Cl_2_]** (**I**) in form of light-violet prisms was obtained by recrystallization of hydrated compounds (55 mg) from a methanol (10 ml) solution. Yield: 40 mg (60%). Analysis calculated for C_16_H_34_Cl_2_CuN_6_O_4_: C 37.76, H 6.73, N 16.51%. Found: C 37.65, H 6.82, N 16.35%.

For the preparation of the complex **[CuCd(*****L*****)(H_2_O)Cl_2_]*****_n_*****·1.25H_2_O** (**II**), a solution of 47 mg (0.2 mmol) Cd(NO_3_)_2_ in 5 ml of ethanol was mixed with 10 ml of aqueous solution containing 109 mg (0.2 mml) of Cu(*H_2_L*)Cl_2_·2H_2_O and refluxed for 2 h. After filtration, the mixture was kept in a refrigerator. A light violet precipitate formed over several days was filtered off, washed with small amounts of methanol and diethyl ether, and dried in air. Yield: 74 mg (56%). Analysis calculated for C_16_H_36.5_ CdCl_2_CuN_6_O_6.25_: C 29.12, H 5.58, N 12.74%. Found: C 29.01, H 5.71, N 12.65%. Single crystals of **I** and **II** suitable for X-ray diffraction analysis were selected from the samples resulting from the syntheses.

## 6. Refinement

Crystal data, data collection and structure refinement details are summarized in Table 5 ▸. The methyl­ene H, secondary amine H atoms in **I** and **II** and water H atoms in **II** were placed in geometrically idealized positions and constrained to ride on their parent atoms with *U*_iso_(H) values of 1.2*U*_eq_(C), 1.2*U*_eq_(N) and 1.5*U*_eq_(O), respectively. The carboxyl­ate H atoms in **I** were positioned geometrically and refined as riding with *U*_iso_(H) = 1.5*U*_eq_(O). Because of low site occupancies the water mol­ecules of crystallization O2*W*–O5*W* in **II** were refined in an isotropic approximation.

## Supplementary Material

Crystal structure: contains datablock(s) I, II. DOI: 10.1107/S2056989025003792/hb8137sup1.cif

Structure factors: contains datablock(s) I. DOI: 10.1107/S2056989025003792/hb8137Isup2.hkl

Structure factors: contains datablock(s) II. DOI: 10.1107/S2056989025003792/hb8137IIsup3.hkl

CCDC references: 2420902, 2420903

Additional supporting information:  crystallographic information; 3D view; checkCIF report

## Figures and Tables

**Table 1:** Selected geometric parameters (Å, °) for **I**[Chem scheme1]

Cu1—Cl1	2.8889 (7)	Cu1—N1	2.000 (2)
Cu1—N3	2.003 (2)		
			
N1—Cu1—N3^i^	85.92 (9)	N1—Cu1—N3	94.08 (9)

Symmetry code: (i) 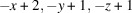.

**Table 2:** Selected geometric parameters (Å, °) for **II**[Chem scheme1]

Cd1—Cl1	2.5125 (18)	Cu1—N4	1.993 (5)
Cd1—Cl2	2.515 (2)	Cu1—N1	2.023 (5)
Cd1—O3^i^	2.266 (4)	Cu1—N6	2.006 (5)
Cd1—O1	2.275 (5)	Cu1—N3	2.020 (5)
Cd1—O4^i^	2.637 (6)	Cu1—O1*W*	2.446 (5)
Cd1—O2	2.494 (6)	Cu1—Cl1^ii^	3.048 (2)
			
Cl1—Cd1—Cl2	104.67 (7)	N4—Cu1—N3	86.5 (2)
O3^i^—Cd1—O4^i^	52.44 (17)	N6—Cu1—N1	86.6 (2)
O1—Cd1—O2	53.54 (19)	N3—Cu1—N1	93.0 (2)
N4—Cu1—N6	93.9 (2)		

Symmetry codes: (i) 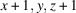; (ii) 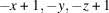.

**Table 3:** Hydrogen-bond geometry (Å, °) for **I**[Chem scheme1]

*D*—H⋯*A*	*D*—H	H⋯*A*	*D*⋯*A*	*D*—H⋯*A*
N1—H1⋯O2^i^	1.00	2.03	2.877 (3)	141
O1—H1*C*⋯Cl1^ii^	0.93 (3)	2.12 (3)	3.030 (2)	168 (3)
N3—H3⋯Cl1^iii^	1.00	2.52	3.381 (2)	144

Symmetry codes: (i) 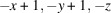; (ii) 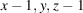; (iii) 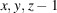.

**Table 4:** Hydrogen-bond geometry (Å, °) for **II**[Chem scheme1]

*D*—H⋯*A*	*D*—H	H⋯*A*	*D*⋯*A*	*D*—H⋯*A*
O1*W*—H1*WB*⋯O3^ii^	0.85	2.06	2.787 (7)	143
N4—H4⋯O4^iii^	0.98	2.08	2.961 (8)	148
N1—H1⋯Cl2^iv^	0.98	2.54	3.377 (5)	143
N6—H6⋯Cl1^i^	0.98	2.77	3.379 (5)	121
N3—H3⋯O2*W*	0.98	2.04	3.006 (13)	167
O1*W*—H1*WA*⋯O2*W*	0.85	2.28	3.037 (14)	149
O2*W*—H2*WA*⋯O2^iv^	0.85	1.93	2.721 (14)	154

Symmetry codes: (i) 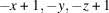; (ii) 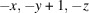; (iii) 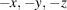; (iv) 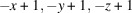.

**Table 5:** Experimental details

	**I**	**II**
Crystal data		
Chemical formula	[CuCl_2_(C_16_H_34_N_6_O_4_)]	[CuCd(C_16_H_32_N_6_O_4_)Cl_2_(H_2_O)]·1.25H_2_O
*M*_r_	508.93	659.85
Crystal system, space group	Monoclinic, *P*2_1_/*c*	Triclinic, *P* 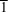
Temperature (K)	100	273
*a*, *b*, *c* (Å)	10.0036 (5), 15.8231 (7), 6.9325 (3)	10.1565 (4), 10.4249 (5), 14.6531 (7)
α, β, γ (°)	90, 99.808 (2), 90	80.305 (3), 80.117 (3), 64.149 (3)
*V* (Å^3^)	1081.29 (9)	1367.82 (11)
*Z*	2	2
Radiation type	Mo *K*α	Mo *K*α
μ (mm^−1^)	1.29	1.79
Crystal size (mm)	0.12 × 0.07 × 0.06	0.15 × 0.05 × 0.05

Data collection		
Diffractometer	Bruker APEXII CCD	Bruker APEXII CCD
Absorption correction	Multi-scan (*CrysAlis PRO*; Rigaku OD, 2022 ▸)	Multi-scan (*CrysAlis PRO*; Rigaku OD, 2022 ▸)
*T*_min_, *T*_max_	0.892, 0.920	0.895, 0.910
No. of measured, independent and observed [*I* > 2σ(*I*)] reflections	5849, 1844, 1509	12964, 4762, 3446
*R*_int_	0.089	0.040
(sin θ/λ)_max_ (Å^−1^)	0.590	0.595

Refinement		
*R*[*F*^2^ > 2σ(*F*^2^)], *wR*(*F*^2^), *S*	0.029, 0.067, 1.05	0.050, 0.149, 1.04
No. of reflections	1844	4762
No. of parameters	136	297
No. of restraints	0	3
H-atom treatment	H atoms treated by a mixture of independent and constrained refinement	H-atom parameters constrained
Δρ_max_, Δρ_min_ (e Å^−3^)	0.30, −0.33	1.31, −0.69

Computer programs: *CrysAlis PRO* (Rigaku OD, 2022 ▸), *SHELXT2018/2* (Sheldrick, 2015*a* ▸), *SHELXL2018/3* (Sheldrick, 2015*b* ▸), *Mercury* (Macrae *et al.*, 2020 ▸) and *publCIF* (Westrip, 2010 ▸).

## supplementary crystallographic information

### [3,10-Bis(3-carboxypropyl)-1,3,5,8,10,12-hexaazacyclotetradecane-κ4N1,N5,N8,N12]dichloridocopper(II) (I) Crystal data

**Table:**

[CuCl_2_(C_16_H_34_N_6_O_4_)]	*F*(000) = 534
*M**_r_* = 508.93	*D*_x_ = 1.563 Mg m^−^^3^
Monoclinic, *P*2_1_/*c*	Mo *K*α radiation, λ = 0.71073 Å
*a* = 10.0036 (5) Å	Cell parameters from 1452 reflections
*b* = 15.8231 (7) Å	θ = 2.0–24.9°
*c* = 6.9325 (3) Å	µ = 1.29 mm^−^^1^
β = 99.808 (2)°	*T* = 100 K
*V* = 1081.29 (9) Å^3^	Prism, clear light violet
*Z* = 2	0.12 × 0.07 × 0.06 mm

### [3,10-Bis(3-carboxypropyl)-1,3,5,8,10,12-hexaazacyclotetradecane-κ4N1,N5,N8,N12]dichloridocopper(II) (I) Data collection

**Table:**

Bruker APEXII CCD diffractometer	1509 reflections with *I* > 2σ(*I*)
φ and ω scans	*R*_int_ = 0.089
Absorption correction: multi-scan (CrysAlis PRO; Rigaku OD, 2022)	θ_max_ = 24.8°, θ_min_ = 2.1°
*T*_min_ = 0.892, *T*_max_ = 0.920	*h* = −11→11
5849 measured reflections	*k* = −18→18
1844 independent reflections	*l* = 0→8

### [3,10-Bis(3-carboxypropyl)-1,3,5,8,10,12-hexaazacyclotetradecane-κ4N1,N5,N8,N12]dichloridocopper(II) (I) Refinement

**Table:**

Refinement on *F*^2^	Primary atom site location: dual
Least-squares matrix: full	Hydrogen site location: inferred from neighbouring sites
*R*[*F*^2^ > 2σ(*F*^2^)] = 0.029	H atoms treated by a mixture of independent and constrained refinement
*wR*(*F*^2^) = 0.067	*w* = 1/[σ^2^(*F*_o_^2^) + (0.0246*P*)^2^ + 0.8397*P*] where *P* = (*F*_o_^2^ + 2*F*_c_^2^)/3
*S* = 1.05	(Δ/σ)_max_ < 0.001
1844 reflections	Δρ_max_ = 0.30 e Å^−^^3^
136 parameters	Δρ_min_ = −0.33 e Å^−^^3^
0 restraints	

### [3,10-Bis(3-carboxypropyl)-1,3,5,8,10,12-hexaazacyclotetradecane-κ4N1,N5,N8,N12]dichloridocopper(II) (I) Special details

**Table:**

Geometry. All esds (except the esd in the dihedral angle between two l.s. planes) are estimated using the full covariance matrix. The cell esds are taken into account individually in the estimation of esds in distances, angles and torsion angles; correlations between esds in cell parameters are only used when they are defined by crystal symmetry. An approximate (isotropic) treatment of cell esds is used for estimating esds involving l.s. planes.
Refinement. Refined as a 2-component twin.

### [3,10-Bis(3-carboxypropyl)-1,3,5,8,10,12-hexaazacyclotetradecane-κ4N1,N5,N8,N12]dichloridocopper(II) (I) Fractional atomic coordinates and isotropic or equivalent isotropic displacement parameters (Å^2^)

**Table:**

	*x*	*y*	*z*	*U*_iso_*/*U*_eq_	
Cu1	1.000000	0.500000	0.500000	0.01515 (14)	
Cl1	1.01968 (7)	0.39266 (4)	0.84077 (10)	0.01969 (18)	
N3	0.9904 (2)	0.40064 (14)	0.3191 (3)	0.0158 (5)	
H3	0.975658	0.422479	0.181720	0.019*	
O1	0.2900 (2)	0.30989 (14)	−0.0055 (3)	0.0279 (5)	
H1C	0.213 (3)	0.338 (2)	−0.068 (4)	0.042*	
O2	0.3928 (2)	0.39295 (14)	−0.1942 (3)	0.0307 (6)	
N1	0.7985 (2)	0.51266 (14)	0.4688 (3)	0.0155 (5)	
H1	0.768622	0.542496	0.341645	0.019*	
N2	0.7465 (2)	0.37665 (15)	0.3054 (3)	0.0176 (5)	
C1	0.7212 (3)	0.43239 (18)	0.4585 (4)	0.0194 (7)	
H1A	0.744468	0.402711	0.585529	0.023*	
H1B	0.623066	0.445666	0.438437	0.023*	
C8	0.3969 (3)	0.33740 (19)	−0.0747 (4)	0.0194 (7)	
C5	0.6976 (3)	0.40643 (19)	0.1045 (4)	0.0195 (7)	
H5A	0.771170	0.437569	0.056237	0.023*	
H5B	0.620731	0.445800	0.104723	0.023*	
C4	0.7700 (3)	0.57019 (18)	0.6243 (4)	0.0183 (6)	
H4A	0.776660	0.539459	0.749953	0.022*	
H4B	0.677474	0.593952	0.589865	0.022*	
C7	0.5245 (3)	0.29165 (19)	0.0142 (4)	0.0211 (7)	
H7A	0.519929	0.232826	−0.035123	0.025*	
H7B	0.529656	0.289377	0.157987	0.025*	
C3	1.1253 (3)	0.35995 (18)	0.3585 (4)	0.0175 (6)	
H3A	1.136479	0.321178	0.250265	0.021*	
H3B	1.135718	0.327019	0.481560	0.021*	
C6	0.6522 (3)	0.3330 (2)	−0.0314 (4)	0.0227 (7)	
H6A	0.635330	0.353432	−0.168322	0.027*	
H6B	0.725710	0.290396	−0.019189	0.027*	
C2	0.8799 (3)	0.33948 (18)	0.3360 (4)	0.0176 (6)	
H2A	0.881701	0.293733	0.239062	0.021*	
H2B	0.897761	0.313604	0.467921	0.021*	

### [3,10-Bis(3-carboxypropyl)-1,3,5,8,10,12-hexaazacyclotetradecane-κ4N1,N5,N8,N12]dichloridocopper(II) (I) Atomic displacement parameters (Å^2^)

**Table:**

	*U*^11^	*U*^22^	*U*^33^	*U*^12^	*U*^13^	*U*^23^
Cu1	0.0137 (2)	0.0147 (2)	0.0171 (3)	−0.0005 (2)	0.0027 (2)	−0.0023 (2)
Cl1	0.0213 (4)	0.0218 (4)	0.0161 (4)	−0.0002 (3)	0.0034 (3)	0.0017 (3)
N3	0.0140 (12)	0.0170 (12)	0.0155 (12)	−0.0008 (10)	−0.0002 (10)	0.0024 (9)
O1	0.0209 (12)	0.0300 (13)	0.0332 (12)	−0.0003 (10)	0.0056 (10)	0.0070 (10)
O2	0.0233 (12)	0.0330 (13)	0.0343 (13)	0.0010 (10)	0.0007 (10)	0.0156 (11)
N1	0.0180 (12)	0.0166 (14)	0.0122 (12)	0.0004 (10)	0.0034 (10)	0.0009 (10)
N2	0.0176 (13)	0.0188 (13)	0.0152 (13)	−0.0019 (11)	−0.0010 (10)	0.0005 (10)
C1	0.0178 (16)	0.0219 (16)	0.0188 (16)	−0.0027 (13)	0.0039 (12)	0.0016 (13)
C8	0.0234 (17)	0.0184 (16)	0.0164 (15)	−0.0037 (13)	0.0032 (13)	−0.0041 (13)
C5	0.0157 (15)	0.0231 (17)	0.0185 (15)	−0.0031 (13)	−0.0005 (12)	0.0040 (12)
C4	0.0146 (15)	0.0230 (16)	0.0177 (16)	0.0051 (13)	0.0036 (12)	−0.0021 (13)
C7	0.0222 (18)	0.0207 (16)	0.0191 (15)	−0.0012 (13)	−0.0006 (13)	0.0000 (13)
C3	0.0205 (16)	0.0183 (16)	0.0133 (14)	0.0069 (12)	0.0021 (12)	−0.0018 (12)
C6	0.0213 (17)	0.0298 (18)	0.0165 (16)	0.0032 (14)	0.0019 (13)	−0.0019 (13)
C2	0.0202 (16)	0.0154 (15)	0.0162 (15)	−0.0023 (13)	0.0004 (12)	0.0015 (12)

### [3,10-Bis(3-carboxypropyl)-1,3,5,8,10,12-hexaazacyclotetradecane-κ4N1,N5,N8,N12]dichloridocopper(II) (I) Geometric parameters (Å, º)

**Table:**

Cu1—Cl1	2.8889 (7)	C1—H1B	0.9900
Cu1—N3	2.003 (2)	C8—C7	1.505 (4)
Cu1—N3^i^	2.003 (2)	C5—H5A	0.9900
Cu1—N1^i^	2.000 (2)	C5—H5B	0.9900
Cu1—N1	2.000 (2)	C5—C6	1.516 (4)
N3—H3	1.0000	C4—H4A	0.9900
N3—C3	1.478 (4)	C4—H4B	0.9900
N3—C2	1.489 (4)	C4—C3^i^	1.513 (4)
O1—H1C	0.93 (4)	C7—H7A	0.9900
O1—C8	1.319 (4)	C7—H7B	0.9900
O2—C8	1.204 (3)	C7—C6	1.515 (4)
N1—H1	1.0000	C3—H3A	0.9900
N1—C1	1.482 (4)	C3—H3B	0.9900
N1—C4	1.475 (4)	C6—H6A	0.9900
N2—C1	1.436 (4)	C6—H6B	0.9900
N2—C5	1.472 (4)	C2—H2A	0.9900
N2—C2	1.441 (4)	C2—H2B	0.9900
C1—H1A	0.9900		
			
N3^i^—Cu1—Cl1	87.71 (7)	N2—C5—H5B	109.4
N3—Cu1—Cl1	92.29 (6)	N2—C5—C6	111.0 (2)
N3^i^—Cu1—N3	180.0	H5A—C5—H5B	108.0
N1^i^—Cu1—Cl1	85.74 (7)	C6—C5—H5A	109.4
N1—Cu1—Cl1	94.26 (7)	C6—C5—H5B	109.4
N1^i^—Cu1—N3^i^	94.08 (9)	N1—C4—H4A	110.3
N1—Cu1—N3^i^	85.92 (9)	N1—C4—H4B	110.3
N1^i^—Cu1—N3	85.92 (9)	N1—C4—C3^i^	106.9 (2)
N1—Cu1—N3	94.08 (9)	H4A—C4—H4B	108.6
N1—Cu1—N1^i^	180.0	C3^i^—C4—H4A	110.3
Cu1—N3—H3	108.0	C3^i^—C4—H4B	110.3
C3—N3—Cu1	106.34 (16)	C8—C7—H7A	108.9
C3—N3—H3	108.0	C8—C7—H7B	108.9
C3—N3—C2	111.7 (2)	C8—C7—C6	113.2 (2)
C2—N3—Cu1	114.69 (17)	H7A—C7—H7B	107.8
C2—N3—H3	108.0	C6—C7—H7A	108.9
C8—O1—H1C	109.5	C6—C7—H7B	108.9
Cu1—N1—H1	106.7	N3—C3—C4^i^	107.1 (2)
C1—N1—Cu1	115.30 (18)	N3—C3—H3A	110.3
C1—N1—H1	106.7	N3—C3—H3B	110.3
C4—N1—Cu1	107.42 (17)	C4^i^—C3—H3A	110.3
C4—N1—H1	106.7	C4^i^—C3—H3B	110.3
C4—N1—C1	113.6 (2)	H3A—C3—H3B	108.6
C1—N2—C5	115.5 (2)	C5—C6—H6A	109.2
C1—N2—C2	114.7 (2)	C5—C6—H6B	109.2
C2—N2—C5	114.8 (2)	C7—C6—C5	112.0 (2)
N1—C1—H1A	108.8	C7—C6—H6A	109.2
N1—C1—H1B	108.8	C7—C6—H6B	109.2
N2—C1—N1	113.9 (2)	H6A—C6—H6B	107.9
N2—C1—H1A	108.8	N3—C2—H2A	108.8
N2—C1—H1B	108.8	N3—C2—H2B	108.8
H1A—C1—H1B	107.7	N2—C2—N3	113.9 (2)
O1—C8—C7	112.1 (3)	N2—C2—H2A	108.8
O2—C8—O1	123.8 (3)	N2—C2—H2B	108.8
O2—C8—C7	124.1 (3)	H2A—C2—H2B	107.7
N2—C5—H5A	109.4		
			
Cu1—N3—C3—C4^i^	−43.6 (2)	C1—N2—C2—N3	−70.1 (3)
Cu1—N3—C2—N2	57.0 (3)	C8—C7—C6—C5	82.0 (3)
Cu1—N1—C1—N2	−56.5 (3)	C5—N2—C1—N1	−67.4 (3)
Cu1—N1—C4—C3^i^	40.6 (2)	C5—N2—C2—N3	67.2 (3)
O1—C8—C7—C6	−168.6 (2)	C4—N1—C1—N2	179.0 (2)
O2—C8—C7—C6	11.0 (4)	C3—N3—C2—N2	178.0 (2)
N2—C5—C6—C7	68.7 (3)	C2—N3—C3—C4^i^	−169.4 (2)
C1—N1—C4—C3^i^	169.3 (2)	C2—N2—C1—N1	69.6 (3)
C1—N2—C5—C6	−146.7 (3)	C2—N2—C5—C6	76.4 (3)

Symmetry code: (i) −*x*+2, −*y*+1, −*z*+1.

### [3,10-Bis(3-carboxypropyl)-1,3,5,8,10,12-hexaazacyclotetradecane-κ4N1,N5,N8,N12]dichloridocopper(II) (I) Hydrogen-bond geometry (Å, º)

**Table:**

*D*—H···*A*	*D*—H	H···*A*	*D*···*A*	*D*—H···*A*
N1—H1···O2^ii^	1.00	2.03	2.877 (3)	141
O1—H1*C*···Cl1^iii^	0.93 (3)	2.12 (3)	3.030 (2)	168 (3)
N3—H3···Cl1^iv^	1.00	2.52	3.381 (2)	144

Symmetry codes: (ii) −*x*+1, −*y*+1, −*z*; (iii) *x*−1, *y*, *z*−1; (iv) *x*, *y*, *z*−1.

### Poly[[aqua[µ3-3,10-bis(3-carboxypropyl)-1,3,5,8,10,12-hexaazacyclotetradecane-κ4N1,N5,N8,N12:κ2O,O':κ2O'',O''']-µ-chloridocopper(II)cadmium(II]] 1.25-hydrate] (II) Crystal data

**Table:**

[CuCd(C_16_H_32_N_6_O_4_)Cl_2_(H_2_O)]·1.25H_2_O	*Z* = 2
*M**_r_* = 659.85	*F*(000) = 671
Triclinic, *P*1	*D*_x_ = 1.602 Mg m^−^^3^
*a* = 10.1565 (4) Å	Mo *K*α radiation, λ = 0.71073 Å
*b* = 10.4249 (5) Å	Cell parameters from 2340 reflections
*c* = 14.6531 (7) Å	θ = 2.5–25.1°
α = 80.305 (3)°	µ = 1.79 mm^−^^1^
β = 80.117 (3)°	*T* = 273 K
γ = 64.149 (3)°	Prism, clear light violet
*V* = 1367.82 (11) Å^3^	0.15 × 0.05 × 0.05 mm

### Poly[[aqua[µ3-3,10-bis(3-carboxypropyl)-1,3,5,8,10,12-hexaazacyclotetradecane-κ4N1,N5,N8,N12:κ2O,O':κ2O'',O''']-µ-chloridocopper(II)cadmium(II]] 1.25-hydrate] (II) Data collection

**Table:**

Bruker APEXII CCD diffractometer	3446 reflections with *I* > 2σ(*I*)
φ and ω scans	*R*_int_ = 0.040
Absorption correction: multi-scan (CrysAlis PRO; Rigaku OD, 2022)	θ_max_ = 25.0°, θ_min_ = 2.5°
*T*_min_ = 0.895, *T*_max_ = 0.910	*h* = −11→12
12964 measured reflections	*k* = −12→12
4762 independent reflections	*l* = −15→17

### Poly[[aqua[µ3-3,10-bis(3-carboxypropyl)-1,3,5,8,10,12-hexaazacyclotetradecane-κ4N1,N5,N8,N12:κ2O,O':κ2O'',O''']-µ-chloridocopper(II)cadmium(II]] 1.25-hydrate] (II) Refinement

**Table:**

Refinement on *F*^2^	Primary atom site location: dual
Least-squares matrix: full	Hydrogen site location: mixed
*R*[*F*^2^ > 2σ(*F*^2^)] = 0.050	H-atom parameters constrained
*wR*(*F*^2^) = 0.149	*w* = 1/[σ^2^(*F*_o_^2^) + (0.0757*P*)^2^ + 2.6254*P*] where *P* = (*F*_o_^2^ + 2*F*_c_^2^)/3
*S* = 1.04	(Δ/σ)_max_ = 0.001
4762 reflections	Δρ_max_ = 1.31 e Å^−^^3^
297 parameters	Δρ_min_ = −0.69 e Å^−^^3^
3 restraints	

### Poly[[aqua[µ3-3,10-bis(3-carboxypropyl)-1,3,5,8,10,12-hexaazacyclotetradecane-κ4N1,N5,N8,N12:κ2O,O':κ2O'',O''']-µ-chloridocopper(II)cadmium(II]] 1.25-hydrate] (II) Special details

**Table:**

Geometry. All esds (except the esd in the dihedral angle between two l.s. planes) are estimated using the full covariance matrix. The cell esds are taken into account individually in the estimation of esds in distances, angles and torsion angles; correlations between esds in cell parameters are only used when they are defined by crystal symmetry. An approximate (isotropic) treatment of cell esds is used for estimating esds involving l.s. planes.
Refinement. Refined as a 2-component twin.

### Poly[[aqua[µ3-3,10-bis(3-carboxypropyl)-1,3,5,8,10,12-hexaazacyclotetradecane-κ4N1,N5,N8,N12:κ2O,O':κ2O'',O''']-µ-chloridocopper(II)cadmium(II]] 1.25-hydrate] (II) Fractional atomic coordinates and isotropic or equivalent isotropic displacement parameters (Å^2^)

**Table:**

	*x*	*y*	*z*	*U*_iso_*/*U*_eq_	Occ. (<1)
Cd1	0.59670 (6)	0.26853 (5)	0.76221 (3)	0.04306 (19)	
Cu1	0.33040 (8)	0.22435 (8)	0.19607 (5)	0.0368 (2)	
Cl1	0.51803 (19)	0.08287 (18)	0.85462 (13)	0.0485 (4)	
Cl2	0.3795 (2)	0.5038 (2)	0.78750 (15)	0.0630 (5)	
O3	−0.2427 (5)	0.2757 (5)	−0.1510 (4)	0.0526 (13)	
N4	0.1402 (5)	0.2125 (6)	0.1942 (4)	0.0387 (13)	
H4	0.163812	0.116420	0.178712	0.046*	
O1	0.6079 (6)	0.2308 (6)	0.6120 (3)	0.0617 (14)	
N1	0.5280 (6)	0.2264 (6)	0.1986 (4)	0.0391 (13)	
H1	0.509753	0.317288	0.220179	0.047*	
O4	−0.1352 (6)	0.0727 (6)	−0.2134 (4)	0.0642 (15)	
N6	0.3616 (5)	0.2651 (5)	0.0573 (4)	0.0365 (12)	
H6	0.402797	0.173055	0.031144	0.044*	
N3	0.3019 (6)	0.1802 (6)	0.3358 (4)	0.0389 (12)	
H3	0.268598	0.269992	0.363502	0.047*	
O1W	0.2008 (7)	0.4777 (6)	0.2217 (4)	0.0706 (16)	
H1WA	0.209328	0.487228	0.276563	0.106*	
H1WB	0.244848	0.523768	0.186423	0.106*	
N2	0.5593 (6)	0.1102 (6)	0.3580 (4)	0.0433 (14)	
N5	0.1150 (6)	0.3123 (6)	0.0317 (4)	0.0455 (14)	
O2	0.7049 (7)	0.3750 (7)	0.6212 (4)	0.0745 (17)	
C16	−0.1341 (8)	0.1573 (8)	−0.1647 (5)	0.0432 (16)	
C12	0.6701 (8)	0.3075 (8)	0.5748 (5)	0.0492 (18)	
C8	0.5961 (7)	0.2262 (8)	0.1016 (5)	0.0489 (18)	
H8A	0.642826	0.129227	0.084057	0.059*	
H8B	0.670876	0.262438	0.095161	0.059*	
C9	0.5517 (7)	0.2254 (8)	0.4074 (5)	0.0446 (16)	
H9A	0.496317	0.223751	0.468128	0.053*	
H9B	0.499763	0.317219	0.372695	0.053*	
C6	0.2278 (7)	0.3611 (7)	0.0110 (5)	0.0445 (16)	
H6A	0.190725	0.456242	0.030616	0.053*	
H6B	0.254370	0.368360	−0.055885	0.053*	
C2	0.4356 (8)	0.0744 (8)	0.3782 (5)	0.0491 (18)	
H2A	0.411541	0.064332	0.445198	0.059*	
H2B	0.463691	−0.017891	0.356448	0.059*	
C1	0.6218 (7)	0.1091 (8)	0.2624 (5)	0.0473 (18)	
H1A	0.641518	0.018262	0.241569	0.057*	
H1B	0.715277	0.115140	0.258505	0.057*	
C7	0.4782 (8)	0.3202 (8)	0.0389 (5)	0.0483 (17)	
H7A	0.437003	0.419018	0.052579	0.058*	
H7B	0.519349	0.315835	−0.025881	0.058*	
C5	0.0440 (7)	0.3178 (8)	0.1249 (5)	0.0469 (17)	
H5A	−0.042463	0.299751	0.126945	0.056*	
H5B	0.011290	0.413532	0.142412	0.056*	
C4	0.0662 (7)	0.2235 (9)	0.2911 (5)	0.0499 (18)	
H4A	−0.009896	0.188734	0.299020	0.060*	
H4B	0.020861	0.322564	0.304598	0.060*	
C13	0.1324 (8)	0.1938 (8)	−0.0182 (5)	0.0501 (18)	
H13A	0.156987	0.106670	0.024350	0.060*	
H13B	0.212635	0.178056	−0.067872	0.060*	
C3	0.1813 (8)	0.1339 (8)	0.3557 (5)	0.0499 (18)	
H3A	0.138471	0.145448	0.419911	0.060*	
H3B	0.218770	0.033436	0.346619	0.060*	
C14	−0.0074 (8)	0.2270 (8)	−0.0587 (6)	0.056 (2)	
H14A	−0.083924	0.232508	−0.007732	0.067*	
H14B	−0.037838	0.320795	−0.094049	0.067*	
C15	0.0027 (8)	0.1229 (8)	−0.1198 (6)	0.0536 (19)	
H15A	0.028909	0.030147	−0.083454	0.064*	
H15B	0.082553	0.114088	−0.168889	0.064*	
C10	0.7015 (8)	0.2104 (10)	0.4194 (5)	0.059 (2)	
H10A	0.756318	0.210913	0.358318	0.070*	
H10B	0.752778	0.117877	0.453587	0.070*	
C11	0.7026 (10)	0.3245 (11)	0.4692 (5)	0.068 (2)	
H11A	0.630509	0.416470	0.445325	0.081*	
H11B	0.798489	0.326495	0.453979	0.081*	
O5W	−0.129 (3)	0.756 (3)	0.3727 (17)	0.075 (7)*	0.25
H5WA	−0.133178	0.717443	0.327227	0.113*	0.25
H5WB	−0.043408	0.754503	0.363557	0.113*	0.25
O4W	−0.215 (3)	0.686 (3)	0.3405 (19)	0.085 (7)*	0.25
H4WA	−0.178747	0.593968	0.349859	0.127*	0.25
H4WB	−0.210637	0.725748	0.386049	0.127*	0.25
O2W	0.1567 (12)	0.4480 (13)	0.4332 (8)	0.074 (3)*	0.5
H2WA	0.212914	0.489423	0.431887	0.111*	0.5
H2WB	0.144304	0.419643	0.491597	0.111*	0.5
O3W	−0.142 (3)	0.610 (3)	0.2733 (19)	0.088 (8)*	0.25
H3WA	−0.167073	0.670086	0.312565	0.132*	0.25
H3WB	−0.167283	0.544546	0.302075	0.132*	0.25

### Poly[[aqua[µ3-3,10-bis(3-carboxypropyl)-1,3,5,8,10,12-hexaazacyclotetradecane-κ4N1,N5,N8,N12:κ2O,O':κ2O'',O''']-µ-chloridocopper(II)cadmium(II]] 1.25-hydrate] (II) Atomic displacement parameters (Å^2^)

**Table:**

	*U*^11^	*U*^22^	*U*^33^	*U*^12^	*U*^13^	*U*^23^
Cd1	0.0545 (3)	0.0486 (3)	0.0367 (3)	−0.0289 (3)	−0.0119 (2)	−0.0047 (2)
Cu1	0.0379 (4)	0.0442 (5)	0.0340 (5)	−0.0223 (4)	−0.0087 (3)	0.0006 (4)
Cl1	0.0588 (10)	0.0398 (9)	0.0498 (11)	−0.0251 (8)	−0.0014 (8)	−0.0045 (8)
Cl2	0.0705 (13)	0.0480 (11)	0.0641 (13)	−0.0222 (10)	−0.0027 (10)	−0.0014 (10)
O3	0.053 (3)	0.054 (3)	0.060 (3)	−0.027 (3)	−0.022 (2)	−0.003 (3)
N4	0.034 (3)	0.040 (3)	0.045 (3)	−0.016 (2)	−0.003 (2)	−0.011 (3)
O1	0.093 (4)	0.072 (4)	0.040 (3)	−0.054 (3)	−0.009 (3)	−0.004 (3)
N1	0.042 (3)	0.049 (3)	0.038 (3)	−0.029 (3)	−0.004 (2)	−0.006 (3)
O4	0.076 (4)	0.062 (3)	0.075 (4)	−0.040 (3)	−0.017 (3)	−0.017 (3)
N6	0.044 (3)	0.033 (3)	0.038 (3)	−0.019 (2)	−0.011 (2)	−0.003 (2)
N3	0.046 (3)	0.046 (3)	0.032 (3)	−0.025 (3)	−0.008 (2)	−0.002 (2)
O1W	0.100 (4)	0.047 (3)	0.067 (4)	−0.032 (3)	−0.015 (3)	−0.004 (3)
N2	0.046 (3)	0.052 (3)	0.038 (3)	−0.024 (3)	−0.014 (3)	−0.003 (3)
N5	0.047 (3)	0.044 (3)	0.047 (4)	−0.015 (3)	−0.015 (3)	−0.008 (3)
O2	0.098 (4)	0.096 (5)	0.056 (4)	−0.061 (4)	−0.003 (3)	−0.025 (3)
C16	0.050 (4)	0.042 (4)	0.042 (4)	−0.023 (4)	−0.011 (3)	−0.001 (3)
C12	0.057 (4)	0.050 (4)	0.047 (4)	−0.025 (4)	−0.010 (4)	−0.012 (4)
C8	0.044 (4)	0.064 (5)	0.049 (4)	−0.031 (4)	−0.001 (3)	−0.013 (4)
C9	0.052 (4)	0.052 (4)	0.038 (4)	−0.026 (3)	−0.008 (3)	−0.008 (3)
C6	0.053 (4)	0.038 (4)	0.045 (4)	−0.019 (3)	−0.016 (3)	−0.001 (3)
C2	0.063 (5)	0.051 (4)	0.047 (4)	−0.034 (4)	−0.023 (4)	0.003 (3)
C1	0.043 (4)	0.054 (4)	0.049 (4)	−0.018 (3)	−0.016 (3)	−0.013 (4)
C7	0.062 (4)	0.059 (5)	0.040 (4)	−0.040 (4)	0.003 (3)	−0.010 (3)
C5	0.043 (4)	0.050 (4)	0.049 (4)	−0.015 (3)	−0.012 (3)	−0.012 (3)
C4	0.044 (4)	0.065 (5)	0.045 (4)	−0.026 (4)	0.002 (3)	−0.016 (4)
C13	0.045 (4)	0.050 (4)	0.050 (4)	−0.011 (3)	−0.017 (3)	−0.007 (4)
C3	0.058 (4)	0.065 (5)	0.038 (4)	−0.039 (4)	0.003 (3)	−0.005 (4)
C14	0.050 (4)	0.054 (5)	0.070 (5)	−0.018 (4)	−0.022 (4)	−0.017 (4)
C15	0.053 (4)	0.051 (4)	0.060 (5)	−0.021 (4)	−0.015 (4)	−0.009 (4)
C10	0.063 (5)	0.089 (6)	0.043 (4)	−0.048 (5)	−0.001 (4)	−0.021 (4)
C11	0.093 (6)	0.106 (7)	0.038 (4)	−0.073 (6)	−0.002 (4)	−0.014 (4)

### Poly[[aqua[µ3-3,10-bis(3-carboxypropyl)-1,3,5,8,10,12-hexaazacyclotetradecane-κ4N1,N5,N8,N12:κ2O,O':κ2O'',O''']-µ-chloridocopper(II)cadmium(II]] 1.25-hydrate] (II) Geometric parameters (Å, º)

**Table:**

Cd1—Cl1	2.5125 (18)	C8—C7	1.513 (10)
Cd1—Cl2	2.515 (2)	C9—H9A	0.9700
Cd1—O3^i^	2.266 (4)	C9—H9B	0.9700
Cd1—O1	2.275 (5)	C9—C10	1.498 (9)
Cd1—O4^i^	2.637 (6)	C6—H6A	0.9700
Cd1—O2	2.494 (6)	C6—H6B	0.9700
Cu1—N4	1.993 (5)	C2—H2A	0.9700
Cu1—N1	2.023 (5)	C2—H2B	0.9700
Cu1—N6	2.006 (5)	C1—H1A	0.9700
Cu1—N3	2.020 (5)	C1—H1B	0.9700
Cu1—O1W	2.446 (5)	C7—H7A	0.9700
Cu1—Cl1^ii^	3.048 (2)	C7—H7B	0.9700
O3—C16	1.265 (8)	C5—H5A	0.9700
N4—H4	0.9800	C5—H5B	0.9700
N4—C5	1.491 (8)	C4—H4A	0.9700
N4—C4	1.486 (8)	C4—H4B	0.9700
O1—C12	1.229 (9)	C4—C3	1.505 (10)
N1—H1	0.9800	C13—H13A	0.9700
N1—C8	1.470 (8)	C13—H13B	0.9700
N1—C1	1.478 (8)	C13—C14	1.510 (9)
O4—C16	1.230 (8)	C3—H3A	0.9700
N6—H6	0.9800	C3—H3B	0.9700
N6—C6	1.487 (8)	C14—H14A	0.9700
N6—C7	1.495 (8)	C14—H14B	0.9700
N3—H3	0.9800	C14—C15	1.479 (10)
N3—C2	1.483 (8)	C15—H15A	0.9700
N3—C3	1.471 (8)	C15—H15B	0.9700
O1W—H1WA	0.8500	C10—H10A	0.9700
O1W—H1WB	0.8500	C10—H10B	0.9700
N2—C9	1.471 (8)	C10—C11	1.501 (10)
N2—C2	1.432 (8)	C11—H11A	0.9700
N2—C1	1.435 (9)	C11—H11B	0.9700
N5—C6	1.416 (9)	O5W—H5WA	0.8500
N5—C5	1.428 (9)	O5W—H5WB	0.8500
N5—C13	1.470 (9)	O4W—H4WA	0.8604
O2—C12	1.242 (8)	O4W—H4WB	0.8605
C16—C15	1.513 (9)	O2W—H2WA	0.8498
C12—C11	1.522 (10)	O2W—H2WB	0.8632
C8—H8A	0.9700	O3W—H3WA	0.8501
C8—H8B	0.9700	O3W—H3WB	0.8501
			
Cl1—Cd1—Cl2	104.67 (7)	C10—C9—H9A	109.2
Cl1—Cd1—O4^i^	84.16 (13)	C10—C9—H9B	109.2
Cl2—Cd1—O4^i^	153.76 (12)	N6—C6—H6A	109.0
O3^i^—Cd1—Cl1	103.44 (14)	N6—C6—H6B	109.0
O3^i^—Cd1—Cl2	101.32 (14)	N5—C6—N6	112.8 (5)
O3^i^—Cd1—O1	134.6 (2)	N5—C6—H6A	109.0
O3^i^—Cd1—O4^i^	52.44 (17)	N5—C6—H6B	109.0
O3^i^—Cd1—O2	90.52 (19)	H6A—C6—H6B	107.8
O1—Cd1—Cl1	103.28 (14)	N3—C2—H2A	108.6
O1—Cd1—Cl2	106.49 (16)	N3—C2—H2B	108.6
O1—Cd1—O4^i^	95.06 (19)	N2—C2—N3	114.7 (6)
O1—Cd1—O2	53.54 (19)	N2—C2—H2A	108.6
O2—Cd1—Cl1	154.86 (14)	N2—C2—H2B	108.6
O2—Cd1—Cl2	92.68 (16)	H2A—C2—H2B	107.6
O2—Cd1—O4^i^	88.2 (2)	N1—C1—H1A	108.7
N4—Cu1—N1	177.4 (2)	N1—C1—H1B	108.7
N4—Cu1—N6	93.9 (2)	N2—C1—N1	114.4 (5)
N4—Cu1—N3	86.5 (2)	N2—C1—H1A	108.7
N4—Cu1—O1W	91.0 (2)	N2—C1—H1B	108.7
N1—Cu1—O1W	91.6 (2)	H1A—C1—H1B	107.6
N6—Cu1—N1	86.6 (2)	N6—C7—C8	107.2 (6)
N6—Cu1—N3	179.1 (2)	N6—C7—H7A	110.3
N6—Cu1—O1W	93.9 (2)	N6—C7—H7B	110.3
N3—Cu1—N1	93.0 (2)	C8—C7—H7A	110.3
N3—Cu1—O1W	86.9 (2)	C8—C7—H7B	110.3
C16—O3—Cd1^iii^	100.6 (4)	H7A—C7—H7B	108.5
Cu1—N4—H4	107.3	N4—C5—H5A	108.9
C5—N4—Cu1	115.2 (4)	N4—C5—H5B	108.9
C5—N4—H4	107.3	N5—C5—N4	113.5 (5)
C4—N4—Cu1	106.6 (4)	N5—C5—H5A	108.9
C4—N4—H4	107.3	N5—C5—H5B	108.9
C4—N4—C5	112.7 (5)	H5A—C5—H5B	107.7
C12—O1—Cd1	97.8 (4)	N4—C4—H4A	110.2
Cu1—N1—H1	107.7	N4—C4—H4B	110.2
C8—N1—Cu1	106.7 (4)	N4—C4—C3	107.7 (5)
C8—N1—H1	107.7	H4A—C4—H4B	108.5
C8—N1—C1	113.5 (5)	C3—C4—H4A	110.2
C1—N1—Cu1	113.3 (4)	C3—C4—H4B	110.2
C1—N1—H1	107.7	N5—C13—H13A	109.5
C16—O4—Cd1^iii^	84.1 (4)	N5—C13—H13B	109.5
Cu1—N6—H6	107.3	N5—C13—C14	110.8 (6)
C6—N6—Cu1	116.0 (4)	H13A—C13—H13B	108.1
C6—N6—H6	107.3	C14—C13—H13A	109.5
C6—N6—C7	112.8 (5)	C14—C13—H13B	109.5
C7—N6—Cu1	105.9 (4)	N3—C3—C4	108.8 (6)
C7—N6—H6	107.3	N3—C3—H3A	109.9
Cu1—N3—H3	107.7	N3—C3—H3B	109.9
C2—N3—Cu1	115.1 (4)	C4—C3—H3A	109.9
C2—N3—H3	107.7	C4—C3—H3B	109.9
C3—N3—Cu1	106.6 (4)	H3A—C3—H3B	108.3
C3—N3—H3	107.7	C13—C14—H14A	108.4
C3—N3—C2	111.9 (5)	C13—C14—H14B	108.4
Cu1—O1W—H1WA	109.1	H14A—C14—H14B	107.5
Cu1—O1W—H1WB	108.8	C15—C14—C13	115.5 (6)
H1WA—O1W—H1WB	104.5	C15—C14—H14A	108.4
C2—N2—C9	116.2 (6)	C15—C14—H14B	108.4
C2—N2—C1	115.4 (5)	C16—C15—H15A	108.2
C1—N2—C9	116.2 (6)	C16—C15—H15B	108.2
C6—N5—C5	117.2 (5)	C14—C15—C16	116.5 (6)
C6—N5—C13	116.7 (6)	C14—C15—H15A	108.2
C5—N5—C13	117.3 (6)	C14—C15—H15B	108.2
C12—O2—Cd1	87.1 (5)	H15A—C15—H15B	107.3
O3—C16—C15	117.2 (6)	C9—C10—H10A	108.5
O4—C16—O3	122.9 (6)	C9—C10—H10B	108.5
O4—C16—C15	119.9 (7)	C9—C10—C11	115.1 (7)
O1—C12—O2	121.5 (7)	H10A—C10—H10B	107.5
O1—C12—C11	119.9 (6)	C11—C10—H10A	108.5
O2—C12—C11	118.5 (7)	C11—C10—H10B	108.5
N1—C8—H8A	109.9	C12—C11—H11A	108.3
N1—C8—H8B	109.9	C12—C11—H11B	108.3
N1—C8—C7	108.9 (6)	C10—C11—C12	115.8 (7)
H8A—C8—H8B	108.3	C10—C11—H11A	108.3
C7—C8—H8A	109.9	C10—C11—H11B	108.3
C7—C8—H8B	109.9	H11A—C11—H11B	107.4
N2—C9—H9A	109.2	H5WA—O5W—H5WB	104.5
N2—C9—H9B	109.2	H4WA—O4W—H4WB	113.5
N2—C9—C10	112.0 (6)	H2WA—O2W—H2WB	103.1
H9A—C9—H9B	107.9	H3WA—O3W—H3WB	104.5
			
Cd1^iii^—O3—C16—O4	−0.8 (8)	O2—C12—C11—C10	162.1 (7)
Cd1^iii^—O3—C16—C15	177.9 (5)	C8—N1—C1—N2	178.0 (5)
Cd1—O1—C12—O2	0.0 (8)	C9—N2—C2—N3	74.3 (7)
Cd1—O1—C12—C11	−178.5 (6)	C9—N2—C1—N1	−71.1 (7)
Cd1^iii^—O4—C16—O3	0.7 (7)	C9—C10—C11—C12	78.6 (10)
Cd1^iii^—O4—C16—C15	−177.9 (7)	C6—N6—C7—C8	171.2 (5)
Cd1—O2—C12—O1	0.0 (8)	C6—N5—C5—N4	−69.7 (8)
Cd1—O2—C12—C11	178.5 (7)	C6—N5—C13—C14	−131.6 (7)
Cu1—N4—C5—N5	55.3 (7)	C2—N3—C3—C4	−165.0 (6)
Cu1—N4—C4—C3	−41.9 (6)	C2—N2—C9—C10	152.8 (6)
Cu1—N1—C8—C7	38.4 (6)	C2—N2—C1—N1	69.9 (8)
Cu1—N1—C1—N2	−60.1 (6)	C1—N1—C8—C7	163.9 (5)
Cu1—N6—C6—N5	−55.0 (6)	C1—N2—C9—C10	−66.4 (8)
Cu1—N6—C7—C8	43.3 (6)	C1—N2—C2—N3	−66.8 (8)
Cu1—N3—C2—N2	55.6 (7)	C7—N6—C6—N5	−177.3 (5)
Cu1—N3—C3—C4	−38.4 (6)	C5—N4—C4—C3	−169.2 (6)
O3—C16—C15—C14	4.1 (11)	C5—N5—C6—N6	69.0 (8)
N4—C4—C3—N3	54.4 (7)	C5—N5—C13—C14	81.8 (8)
O1—C12—C11—C10	−19.3 (12)	C4—N4—C5—N5	177.9 (6)
N1—C8—C7—N6	−55.6 (7)	C13—N5—C6—N6	−77.7 (7)
O4—C16—C15—C14	−177.2 (7)	C13—N5—C5—N4	76.8 (7)
N2—C9—C10—C11	179.9 (6)	C13—C14—C15—C16	−177.4 (7)
N5—C13—C14—C15	173.1 (7)	C3—N3—C2—N2	177.4 (6)

Symmetry codes: (i) *x*+1, *y*, *z*+1; (ii) −*x*+1, −*y*, −*z*+1; (iii) *x*−1, *y*, *z*−1.

### Poly[[aqua[µ3-3,10-bis(3-carboxypropyl)-1,3,5,8,10,12-hexaazacyclotetradecane-κ4N1,N5,N8,N12:κ2O,O':κ2O'',O''']-µ-chloridocopper(II)cadmium(II]] 1.25-hydrate] (II) Hydrogen-bond geometry (Å, º)

**Table:**

*D*—H···*A*	*D*—H	H···*A*	*D*···*A*	*D*—H···*A*
O1*W*—H1*WB*···O3^iv^	0.85	2.06	2.787 (7)	143
N4—H4···O4^v^	0.98	2.08	2.961 (8)	148
N1—H1···Cl2^vi^	0.98	2.54	3.377 (5)	143
N6—H6···Cl1^ii^	0.98	2.77	3.379 (5)	121
N3—H3···O2*W*	0.98	2.04	3.006 (13)	167
O1*W*—H1*WA*···O2*W*	0.85	2.28	3.037 (14)	149
O2*W*—H2*WA*···O2^vi^	0.85	1.93	2.721 (14)	154

Symmetry codes: (ii) −*x*+1, −*y*, −*z*+1; (iv) −*x*, −*y*+1, −*z*; (v) −*x*, −*y*, −*z*; (vi) −*x*+1, −*y*+1, −*z*+1.
